# Insights into the Prognostic Role of Serum Interleukin-6 and Hematobiochemical Alterations in Cattle during Recent Outbreaks of Lumpy Skin Disease in Lodhran District, Pakistan

**DOI:** 10.3390/vaccines11010113

**Published:** 2023-01-03

**Authors:** Waqas Ahmad, Muhammad Abu Bakr Shabbir, Mehmood Ahmad, Muhammad Ovais Omer, Rana Muhammad Zahid Mushtaq, Sadaf Aroosa, Asif Iqbal, Arfa Majeed

**Affiliations:** 1 Department of Pathology, University of Veterinary and Animal Sciences Lahore, Lahore 54000, Pakistan; 2 Institute of Microbiology, University of Veterinary and Animal Sciences Lahore, Lahore 54000, Pakistan; 3 Department of Pharmacology and Toxicology, University of Veterinary and Animal Sciences Lahore, Lahore 54000, Pakistan; 4 Department of Pharmacology, Riphah International University, Lahore 54000, Pakistan; 5 Department of Pharmacology, Lords College of Pharmacy Lahore, Lahore 54000, Pakistan; 6 Department of Parasitology, Riphah International University, Lahore 54000, Pakistan

**Keywords:** lumpy skin disease (LSD), LSDV, IL-6, ROC

## Abstract

Lumpy skin disease (LSD) is a highly infectious disease of cattle caused by a virus of the Poxviridae family, genus Capripoxvirus. The present study was designed to determine the prognostic ability of serum IL-6 in LSD using a binary logistic regression model at baseline sampling. A 17-day cohort study was conducted on a recent outbreak of LSD among cattle in the Lodhran District of Punjab, Pakistan. Infected cattle were divided into two categories based on their clinical status on day 17 as recovered (*n* = 33) or unrecovered (*n* = 17). Nodular lesions and scab specimens (*n* = 50) were used for the isolation of the lumpy skin disease virus and were confirmed by PCR. In recovered animals, hematological results showed marked leukocytosis, eosinophilia, lymphocytosis, neutrophilia, and monocytopenia. However, marked erythrocytosis, leukopenia, and thrombocytopenia were observed in the unrecovered animals at the final sampling point of the study. Serum levels of total protein, albumin, and glucose were significantly higher in the recovered animals. Meanwhile, aspartate aminotransferase, alkaline phosphatase, lactate dehydrogenase, creatinine phosphokinase, total bilirubin, and direct bilirubin were found considerably higher in the unrecovered group. Receiver-operating characteristic curve analysis for serum IL-6 at baseline predicts the extended clinical conditions at the cut-off value of 85.16 pg/mL (55% specificity, 94% sensitivity, area under the curve 0.8039, respectively). In conclusion, the disease-induced hematological and biochemical alterations were significantly ameliorated in the recovered animals. In addition, the study revealed that serum IL-6 can be used as a valid marker for predicting the clinical worsening of LSD in cattle.

## 1. Introduction

Lumpy skin disease (LSD) is a transboundary viral disease caused by the lumpy skin disease virus (LSDV), a member of the Poxviridae family, genus Capripoxvirus [1,2]. This virus can cause infection in large ruminants, particularly cattle and buffaloes [3,4]. LSD is characterized by fever, nasal discharge, lacrimation, hypersalivation, anorexia, and lethargy, followed by nodular lesion development in the skin and mucous membranes of the entire body [5,6].

The disease is economically significant since it can result in a significant decrease in milk production, temporary or long-lasting infertility in bulls, damage to hides, and secondary bacterial infections that result in severe clinical signs [7]. LSDV is transmitted either through direct contact (secretions of infected animals) or indirect contact (mosquitoes, ticks, and flies) [8]. The infection produces pronounced hematological, biochemical, and immunological changes [9]. Acute LSD is marked by significant epidermal necrosis, folliculitis, and vasculitis affecting cutaneous blood vessels [10]. LSDV outbreaks often manifest as epidemics [11]. There are frequently inexplicable intervals of several years between epidemics, regardless of humidity, season, or vector abundance [2].

Cytokines perform essential roles in the immune system of dairy cattle by regulating several cellular and physiological activities. In addition, they serve as cell communication transmitters in the inflammatory and immunological responses [12,13].

Interleukin-6 (IL-6), known as a multifunctional cytokine, is a glycoprotein generated and secreted by lymphocytes that activates macrophages and enhances proinflammatory response [14]. The prognostic aspect of IL-6 has been investigated in cattle in several studies [15,16,17]. However, limited information is available regarding serum IL-6 levels in the case of LSD. Therefore, the present study was designed to evaluate the prognostic value of IL-6 regarding the progression of disease along with hematological and biochemical changes induced by LSDV in cattle populations in and around Lodhran district, Pakistan. To determine the precision of IL-6 assessment in serum for prognosis assessment, we performed receiver-operating characteristic (ROC) analysis with the area under the curve (AUC).

## 2. Materials and Methods

### 2.1. Study Area

In present study, a 17-day cohort study was conducted on fifty LSDV-affected cattle in and around Lodhran District, Pakistan. The individual animals were observed at day 0 for clinical and homeostatic alterations (e.g., hematological, clinical chemistry) caused by LSD. In order to determine the prognostic potential, diseased cattle were divided into two categories based on their status on day 17 as recovered or unrecovered.

The samples were taken within and around the Lodhran District, Punjab, Pakistan (29.5339, 71.63244), including cattle farms and several village dairy units. Before the sampling, the first symptoms of the disease were recorded approximately three months earlier.

### 2.2. Animal Population and Clinical Presentation

Physical examination of the affected animals was conducted and documented. The farmers reported that infected animals exhibited distinctive hyperthermia and an initial decline in milk production. The infected animals exhibited nasal discharge, anorexia, lacrimation, emaciation, lymph node enlargement, cutaneous lesions, and buccal mucous membranes. In addition, cutaneous nodules were observed at neck, legs, and back regions. Importantly, LSD-recovered animals from the same diseased farm displayed a lesser extent of symptoms and diminished skin lesions [18].

### 2.3. Samples Collection and Preparation

The nodular lesion swabs were collected in 3 mL of sterile phosphate buffer saline and transported to the lab in ice, according to the previously described method [19]. From each animal, at least two scab samples were taken. The samples (scabs) were ground into a 10% suspension in phosphate buffer saline, filtered in a 0.45 μM syringe filter, and stored at −80 °C until further use.

The animals were sampled on the initial day of observable symptoms and again at 17 days, as described previously [19], with some modifications. After sampling, the animals were further distributed into two subgroups: the recovered group (*n* = 33) consisted of cattle that had clinically recovered 15 to 17 days after the onset of symptoms, and the unrecovered group (*n* = 17) consisted of cattle that had been determined to be unrecovered because they were still exhibiting clinical signs, and still receiving medical treatment, or displayed other abnormalities during the clinical examination. The same veterinarian conducted all clinical examinations to avoid examiner bias.

Blood samples with and without ethylene diamine tetraacetic acid (EDTA) were collected via traumatic jugular venipuncture from the animals. Blood samples added with EDTA were subjected to hematological analysis in an automated hematology analyzer (Beckman Coulter, Brea, CA, USA) while the samples without EDTA were subjected to centrifuged (Eppendorf 5424 R, Darmstadt, Germany) at 3000 rpm for 15 min for serum isolation and kept at −20 °C for biochemical investigations. All samples were collected with the owners’ permission and adhered to the College of Veterinary Sciences, Riphah International University, Lahore guidelines (Rcvets-1038).

### 2.4. Identification of LSDV through PCR

Nodular lesions and scab samples were homogenized in phosphate-buffered saline (50%) after trituration and reconstituted into 10% suspension [11]. The viral DNA was extracted by GeneJET Viral DNA/RNA Purification Kit (Thermo Fisher Scientific, Waltham, MA, USA) per the manufacturer’s instructions. Primer sets designed for the RPO30 gene (F = 5′-TCTATGTCTTGATATGTGGTGGTAG-3′ and R = 5′- AGTGATTAGGTGGTGTATTATTTTCC -3′), encoding a viral attachment protein, were used, as described by [20]. A reaction volume of 25 µL was added with 1 µL of both primers at a concentration of 100 pmol of each primer, 4 µL of DNA template, 12.5 µL of master mix, and up to 25 µL of nuclease-free sterile double-distilled water. For each reaction, both negative and positive controls were added. The following conditions were used for the amplification: initial denaturation cycle at 95 °C for 4 min, followed by 35 cycles (denaturation at 95 °C for 45 s, annealing at 55 °C for 45 s, and extension at 72 °C for 45 s) and a final extension cycle at 72 °C for 10 min, as described previously [21]. PCR products (5 µL) were separated at 90 V for 30 min on a 1% agarose gel using the 100 bp DNA marker (Thermo Scientific). The PCR products were visualized using a gel documentation system (Omega Flour^Plus^, Sanfrancisco, CA, USA). Positive samples were anticipated to generate an amplicon with a 172 bp band size.

### 2.5. Hematological Parameters

Red blood cells (RBCs), hemoglobin (Hb), packed cell volume (PCV), mean corpuscular volume (MCV), mean corpuscular hemoglobin concentration (MCHC), platelets, and white blood cells (WBCs) were estimated in EDTA-containing blood samples using an automatic hematology analyzer. Blood films fixed in (95%) absolute methanol were stained in Giemsa stain for differential leukocyte counts according to the previously described method [22].

### 2.6. Biochemical Parameters and IL-6 Estimation

Serum samples were tested for albumin and total proteins, as documented by Doumas [23] and Drupt [24]. Albumin content was subtracted from total protein concentration to calculate the serum globulin concentration [25]. The aspartate aminotransferase (AST) and alkaline phosphatase (ALP) activity were assessed according to the previously described procedures [26,27]. Lactate dehydrogenase (LDH) was assessed using the method documented in previous study [28]. Creatinine phosphokinase (CPK) was calculated as described in previous study [29]. Creatinine concentration was evaluated according to the previously reported method [30]. Total and direct bilirubin were also calculated according to the previously described method [31], while amount of indirect bilirubin was determined by deducting the amount of direct bilirubin from the total bilirubin. Serum glucose was analyzed according to previously described method [32]. IL-6 was measured using a commercial ELISA kit (Wuhan Fine Biotech Co., Wuhan, China) under the protocol outlined in standard kits.

### 2.7. Statistical Analysis

Excel 2016 software was used to process the experimental findings initially. Regarding the hematobiochemical indices and IL-6 values, data from recovered and unrecovered animals were compared at initial and final sampling times using GraphPad Prism 9. A two-way ANOVA was used to assess group interaction (recovered, unrecovered) and sampling time points (baseline and final).

A binary logistic regression was conducted to investigate the odds of serum IL-6 levels changing at the onset of LSD symptoms (baseline sampling) to forecast a persistent disease progression (unrecovered) using Minitab21. A Hosmer–Lemeshow test was analyzed to evaluate the model’s fit. The influence of the variable IL-6 was assessed using the Wald statistic. Furthermore, to assess the prognostic significance of serum IL-6 concentration, a ROC curve was constructed using the values of recovered and unrecovered animals at the baseline sampling. The AUC was computed, P 0.05 was considered statistically significant [19].

## 3. Results

### 3.1. Confirmation of LSDV through PCR

**Figure 1:**
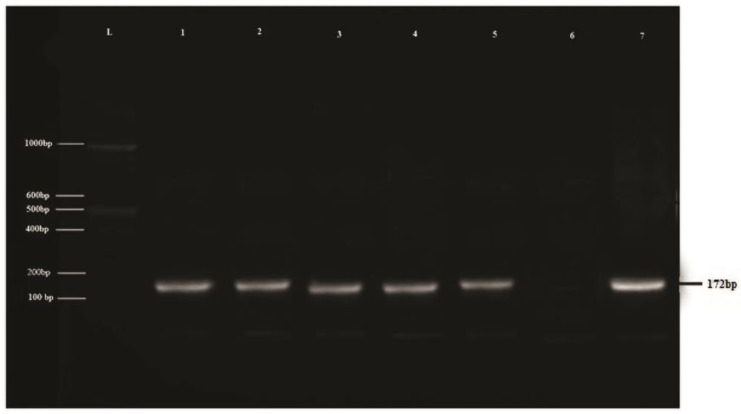
**Agarose gel electrophoresis pattern of RPO30 gene-based PCR assay for detection of LSDV.** L: molecular ladder marker of 100 bp; lanes 1–5: 172-bp RPO30 gene-amplified PCR products from field samples; lane 6: negative control; lane 7: positive control.

A positive PCR amplicon (172 bp) of the RPO30 gene for LSDV was identified through agarose gel electrophoresis. DNA extracted from different LSD clinical samples yields a clear band in the gel electrophoresis after amplifying the target gene (Figure 1).

### 3.2. Hematological Parameters

Hematological findings for recovered and unrecovered animals at both sampling intervals are presented in Table 1.

It was revealed that compared to recovered animals, unrecovered animals presented a significant increase (*p* ≤ 0.05) in hematological indices (RBCs, PCV and Hb). Both groups revealed a significant increase in these values at the final sampling compared to the baseline sampling. A significant decline (*p* ≤ 0.05) in MCV was observed in both groups at final sampling compared to the baseline. Platelet count significantly decreased (*p* ≤ 0.05) in the unrecovered group, while the recovered animals showed a significant increase in the values at the final sampling. Recovered animals showed significant amelioration in hematological indices compared with unrecovered cattle. A marked increase in the TLC, eosinophils, and lymphocytes and a significant decrease in neutrophils and monocytes was observed in recovered animals with the progression of the disease. On the other hand, the final values regarding TLC, neutrophil, eosinophil, lymphocyte, and monocyte were significantly decreased (*p* ≤ 0.05) compared with the initial values in the unrecovered animals. Moreover, TLC and the DLC values were considerably lower (*p* ≤ 0.05) in the unrecovered compared with the recovered animals.

### 3.3. Biochemical Findings

The biochemical changes observed for recovered and unrecovered cattle are shown in Table 2.

Serum levels of total protein, albumin, and glucose were significantly higher in the recovered animals. However, levels of total bilirubin, direct bilirubin, LDH, CPK, AST, and ALP were recorded considerably higher (*p* < 0.05) in the individuals of the unrecovered group compared with the recovered cattle. In unrecovered cattle, total protein and globulin concentrations were considerably decreased (*p* < 0.05).

### 3.4. Serum Interleukin-6 Levels

**Figure 2:**
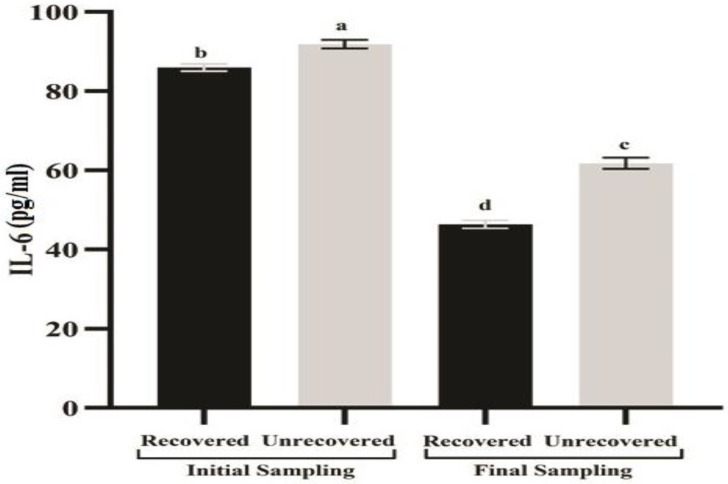
**Comparison of serum IL-6 concentration in LSDV infected cattle.** Means (±SEM) without a common superscript letter differ significantly (*p* < 0.05). The infected cattle were grouped by recovery at 17 days after baseline sampling. The serum IL-6 concentrations of the recovered group (*n* = 33) and the unrecovered group (*n* = 17) were analyzed by two-way ANOVA and the Tukey test.

In the context of disease progression, the unrecovered cattle revealed significantly higher levels of IL-6 mean value (92 ± 1.1 pg/mL) at baseline sampling compared with the recovered cattle (86 ± 0.92 pg/ mL). Moreover, at final sampling, the values were 62 ± 1.5 pg/mL and 33 ± 0.88 pg/mL for unrecovered and recovered cattle, respectively (Figure 2). The binary logistic regression analysis regarding serum IL-6 showed statistical significance, as the results for the P-value of the Hosmer–Lemeshow test were nonsignificant (*p* = 0.225). The Wald statistic showed a significant impact of serum IL-6 concentration in the regression model (*p* = 0.002). The likelihood (odds ratio 1.26; 95% CI 1.0902, 1.4510) of LSD persistence in cattle increased by 26% for each unit of increase in serum IL-6. The prognostic quality of serum IL-6 concentration (Figure 2) regarding the clinical consequence of infection was calculated in a ROC analysis.

**Figure 3:**
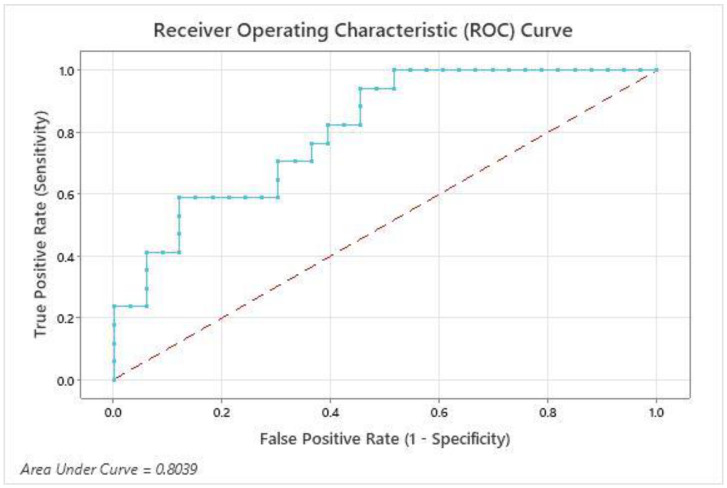
**ROC graph for the estimation of the prognostic role of serum IL-6 in LSDV-infected cattle.** Infected cattle (*n* = 50) were categorized based on clinical recovery status at 17 days after the onset of disease (recovered group, *n* = 33; unrecovered group, *n* = 17).

The ROC analysis for serum IL-6 concentration of the LSDV-infected cattle at the baseline sampling showed a cut-off value of 85.16 pg/mL at a sensitivity of 94% and specificity of 55% for recognizing the cattle with a continued disease duration. The AUC of 0.8039 (0.6834 to 0.9244: 95% CI, *p* = 0.0005) showed a good prognostic ability of serum IL-6 to differentiate between recoverable and unrecoverable animals at the initiation of LSDV infection (Figure 3).

## 4. Discussion

LSD is considered a transboundary livestock disease due to its ability to spread to neighboring countries. It inflicts a considerable impact on trade and food security [33]. This study provides the first insights into the disease-induced hematobiochemical alterations and the prognostic role of IL-6 in LSDV infection from field conditions in Pakistan.

LSDV was diagnosed based on PCR results and clinical symptoms in this investigation. PCR is the most effective method for accurately identifying the infectious agent. PCR demonstrated high sensitivity for screening the LSDV genome in skin nodules. The results are as per the findings of previously reported studies [3,34]. The elevated PCR sensitivity may be due to the tissue tropism of the virus for cutaneous tissues and its ability to remain in high concentrations on the skin [18].

The infected animals reveal leucopenia, lymphopenia, monocytopenia, and eosinopenia, which indicate viral infections [32]. Lymphopenia is also induced by marked corticosteroid hormone production [33]. The findings of present study are in line with previous study findings [9,18]. Thrombocytopenia is mainly attributable to a shortened platelet lifespan in the infection and was observed in many cattle in previous studies [18]. Systemic vasculitis most often triggers this condition due to LSDV’s affinity for endothelial cells, resulting in excessive platelet consumption [35]. 

The serum concentration of total protein and globulin levels were increased in recovered animals. However, dehydration may also increase the total protein level. Globulin concentration is related to the body’s immunological reaction to disease [36]. The reduced serum concentrations of total proteins and albumin in the unrecovered cattle are caused mainly by liver damage, increased protein catabolism, or both [37]. Significantly higher levels of bilirubinemia were observed in the unrecovered animals. This finding could be due to the degenerated hepatocytes adjacent to the bile duct.

Furthermore, intrahepatic cholestasis and biliary may also aggravate the condition [38]. Significantly increased AST levels were observed in the unrecovered individuals, which depict hepatocyte damage, though the injury is subclinical [18]. Additionally, elevated AST also describes cardiac and skeletal muscle injury. Consequently, increased AST in this study might be related to cardiac muscle and skeletal muscle damage due to LSDV. Similar findings have been observed in previous studies [18,38]. It is further confirmed by the elevated LDH and CPK levels in the serum [39]. Serum ALP was significantly raised in unrecovered animals, and this condition may be attributed to the inflammation of biliary duct cells and cholesteric hepatitis [38,39,40]. It is further confirmed by elevated direct bilirubin [41]. Unrecovered cattle in the present study exhibited reduced glucose levels, which may be suggestive of increased glucose catabolism and decreased feed intake during viral infection [18].

The primary sources of IL-6 are macrophages and monocytes. The essential role of IL-6 is B- and T-cell activation and initiating the acute phase response [42]. IL-6 is the primary responding cell factor during acute inflammation to induce C-reactive protein (CRP), serum amyloid A, hepcidin, and fibrinogen production in hepatocytes [43,44]. IL-6 has an extended plasma half-life compared with IL-1β or TNF-α. It is more consistently quantifiable in plasma than the other two cytokines. Some studies have shown a remarkable elevation in IL-1β, IL-6, IFN-γ, and TNF-α cytokines in systemic protozoal diseases in cattle. Its primary role as a biomarker seems to be prognostic instead of diagnostic [43,44,45]. A correlation between elevated serum concentrations of IL-6 and mortality rate has been reported in systemic infections in humans and dogs [43,44,45]. The findings are also consistent with an elevated IL-6 gene expression in intestinal epithelial cells and peripheral blood mononuclear cells from scouring cattle with BRV [19,46].

The present study explored the prognostic value of the serum IL-6 concentration regarding LSDV-affected cattle. The key result was that the cattle developing an extended duration of infection showed considerably elevated IL-6 concentration at the initial symptoms. The binary logistic regression model presented a significant effect of serum IL-6 concentration in forecasting an increased duration of infection. The odds of the continued duration of infection increased by 26% for each unit increase in concentrations of serum IL-6. An ROC analysis further estimated the serum concentration of IL-6 as an initial factor for the prognosis of infected cattle. The AUC evaluated the accuracy of serum IL-6 concentration in distinguishing recovered from unrecovered animals. The value of AUC (0.8039) established the serum IL-6 as a good marker for assessing the prognosis.

Furthermore, the cut-off value was set at 85.16 pg/mL, showing a sensitivity of 94% and specificity of 55% for identifying cattle with a prolonged duration of infection. The results of the ROC analysis and the binary logistic regression revealed that the estimation of IL-6 at the onset of infection was an appreciated tool for recognizing cattle developing a prolonged duration of the disease. It is recommended that cattle exhibiting serum IL-6 concentrations above 85.16 pg/mL should be given attention, as they are susceptible to developing a prolonged infection duration.

In conclusion, the present study revealed serum IL-6 as a valid marker for estimating the progression of LSD in affected cattle. Elevated serum IL-6 concentration could lead the veterinary physician to closely monitor LSDV-infected cattle and adapt the supportive therapy accordingly.

## Figures and Tables

**Table 1:** Values of hematological parameters of the infected cattle at baseline and final sampling. Cattle recovered from LSD at the final sampling were compared with those that did not recover using two-way ANOVA. Means (±SEM) without a common superscript letter differ significantly (*p* < 0.05).

Parameters	First Sampling Time Point		Second Sampling Time Point	
	Recovered Group (*n* = 33)	Unrecovered Group (*n* = 17)	Recovered Group (*n* = 33)	Unrecovered Group (*n* = 17)
RBCs × 10^6^/µL	5.2 ± 0.0047 ^c^	6.6 ± 0.0076 ^a^	5.5 ± 0.0049 ^b^	6.5 ± 0.0072 ^a^
Hb gm%	8.7 ± 0.0054 ^c^	9 ± 0.0087 ^b^	9.5 ± 0.0064 ^a^	9.1 ± 0.011 ^b^
PCV %	29 ± 0.019 ^d^	30 ± 0.035 ^c^	31 ± 0.021 ^b^	31 ± 0.056 ^a^
MCV fl	55 ± 0.063 ^b^	46 ± 0.081 ^d^	56 ± 0.068 ^a^	48 ± 0.11 ^c^
MCHC %	30 ± 0.027 ^a^	30 ± 0.032 ^b^	30 ± 0.024 ^a^	29 ± 0.059 ^c^
Platelets × 10^3^/µL	130 ± 0.15 ^b^	100 ± 0.23 ^c^	140 ± 0.22 ^a^	83 ± 0.41 ^d^
TLC × 10^3^/µL	9.5 ± 0.021 ^b^	7.2 ± 0.038 ^c^	9.8 ± 0.025 ^a^	4.2 ± 0.044 ^d^
Neutrophil × 10^3^/µL	3.8 ± 0.0083 ^a^	3.5 ± 0.0054 ^b^	3.5 ± 0.009 ^c^	2.9 ± 0.0057 ^d^
Eosinophil × 10^3^/µL	0.15 ± 0.00033 ^b^	0.11 ± 0.00059 ^c^	0.16 ± 4 × 10^−4 a^	0.067 ± 7 × 10^−4 d^
Lymphocyte × 10^3^/µL	5.2 ± 0.011 ^b^	3.4 ± 0.038 ^c^	5.9 ± 0.015 ^a^	1.1 ± 0.042 ^d^
Monocyte × 10^3^/µL	0.29 ± 0.00063 ^a^	0.19 ± 0.001 ^c^	0.24 ± 0.00063 ^b^	0.11 ± 0.0012 ^d^

**Table 2:** Results of biochemical parameters of the infected cattle at baseline and final sampling. Cattle recovered from LSD at the final sampling were compared with those that did not recover using two-way ANOVA followed by Tukey test. Means (±SEM) without a common superscript letter differ significantly (*p* < 0.05).

Parameters	First Sampling Time Point		Second Sampling Time Point	
	Recovered Group (*n* = 33)	Unrecovered Group (*n* = 17)	Recovered Group (*n* = 33)	Unrecovered Group (*n* = 17)
Total protein (gm/dL)	5.5 ± 0.0026 ^c^	6.5 ± 0.0037 ^b^	6.9 ± 0.0026 ^a^	6.5 ± 0.0031 ^b^
Albumin (gm/dL)	2.9 ± 0.0034 ^b^	2.7 ± 0.019 ^c^	3 ± 0.014 ^a^	2.7 ± 0.022 ^c^
Globulin (gm/dL)	2.6 ± 0.004 ^b^	3.9 ± 0.02 ^a^	3.9 ± 0.015 ^a^	3.9 ± 0.023 ^a^
T. Bilirubin (mg/dL)	0.26 ± 0.0015 ^d^	0.42 ± 0.0027 ^b^	0.29 ± 0.0015 ^c^	0.52 ± 0.0027 ^a^
D. Bilirubin (mg/dL)	0.24 ± 0.00049 ^c^	0.29 ± 0.003 ^b^	0.23 ± 0.00049 ^c^	0.33 ± 0.001 ^a^
In. Bilirubin (mg/dL)	0.029 ± 0.0016 ^d^	0.13 ± 0.0041 ^b^	0.05 ± 0.0015 ^c^	0.19 ± 0.0027 ^a^
AST (U/L)	54 ± 0.17 ^d^	80 ± 0.72 ^b^	61 ± 0.11 ^c^	89 ± 1.4 ^a^
ALP (U/L)	67 ± 0.2 ^d^	77 ± 0.27 ^b^	68 ± 0.19 ^c^	81 ± 0.49 ^a^
LDH (U/L)	1800 ± 0.65 ^d^	2000 ± 0.89 ^b^	1800 ± 0.61 ^c^	2800 ± 0.83 ^a^
CPK (U/L)	82 ± 0.047 ^d^	89 ± 0.067 ^c^	93 ± 0.058 ^b^	120 ± 0.1 ^a^
Glucose (mg/dL)	62 ± 0.042 ^a^	50 ± 0.038 ^b^	44 ± 0.026 ^c^	43 ± 0.033 ^d^
Creatinine (mg/dL)	1.2 ± 0.0015 ^a^	1.2 ± 0.00082 ^c^	1.2 ± 0.0016 ^b^	1.2 ± 0.00076 ^c^

## Data Availability

All the data are available.

## Abbreviations

Lumpy skin disease	(LSD)
Interleukin-6	(IL-6)
Polymerase chain reaction	(PCR)
Lumpy skin disease virus	(LSDV)
Receiver-operating characteristic	(ROC)
Area under the curve	(AUC)
Ethylene diamine tetraacetic acid	(EDTA)
DNA	(deoxyribonucleic acid)
RNA	(Ribonucleic acid)
Red blood cells	(RBCs)
Hemoglobin	(Hb)
Packed cell volume	(PCV)
Mean corpuscular volume	(MCV)
Mean corpuscular hemoglobin concentration	(MCHC)
White blood cells	(WBCs)
Aspartate aminotransferase	(AST)
Alkaline phosphatase	(ALP)
Lactate dehydrogenase	(LDH)
Creatinine phosphokinase	(CPK)
ELISA	(Enzyme Linked Immunosorbent Assay)
ANOVA	(Analysis of Variance)
TLC	(Total Leukocyte Count)
DLC	(Differential Leukocyte Count)
CPK	(Creatinine Phosphokinase)
TNF-α	(Tumor Necrosis Factor-alpha)
IL-1β	(Interleukin-1 beta)
IFN-γ	(*Interferon gamma)*
Packed cell volume	(PCV)
Confidence interval	(CI)
Picogram per milliliter	(pg/mL)
